# Compound Comminuted Fracture of the Cranium

**Published:** 1880-05

**Authors:** 


### Compound Comminuted Fracture of the Cranium.

James King, enterprising newsboy, walked into the Hospital, June 12, 1879; age 14; American; his mother accompanied him and said his head had been injured, she thought not badly; examination brought to light a fractured skull; a brick had fallen some forty feet, striking upon the upper portion of the occipital bone, as he was stooping over. Dr. Gay was summoned, and elevated and removed 9 fragments of bone from the wound, under ether, leaving a clean cut circular hole in cranium one and one-half inches in diameter; Dura Mater shone beneath uninjured; the boy was not, nor had he been, at all unconscious; after operation wound was stitched up, and cold water compress applied.

June 13. Patient a little delirious; takes milk and beef tea.

June 15. Has the sort of delirium called muttering; wound bleeds.

Junes 16. Removed stitches, giving egress to a quantity of coagulated blood which had collected under the closed lip of the wound, and had been pressing upon the cerebrum; consciousness and sanity soon returned as expected.

June 20. Wound healthy and healing slowly; goes out doors and has returned to the various amusements known to boyhood; pulsation of posterior lobe visible under membrane which has grown over opening.

Aug. 2, 1879. Discharged, recovered, and is as astute and vociferous as formerly.

